# Animating inter-organisational resilience communication: A participatory social network analysis of water governance in the UK

**DOI:** 10.1016/j.heliyon.2020.e05069

**Published:** 2020-10-03

**Authors:** S. Ward, F. Meng, S. Bunney, K. Diao, D. Butler

**Affiliations:** a Centre for Water Systems, College of Engineering, Physical Sciences and Mathematics, University of Exeter, Kay Building, North Park Road, Exeter, EX4 4QF, UK; b Faculty of Technology, De Montfort University, Mill Lane, Leicester, LE2 7DR, UK

**Keywords:** Animation, Governance, Policy, Resilience, Social network analysis, Water, Data visualization, Green engineering, Natural resource management, Network analysis, Knowledge representation

## Abstract

Resilience as a concept and resilience assessment as a practice are being explored across a range of social, ecological and technical systems. In this paper, we propose a new method and visualisation approach for interrogating the communication of resilience within organisational networks, using participatory social network analysis and message passing. Through an examination of the UK water sector organisational network, represented by multiple co-produced network graphs, we identify organisations having a key role in the communication of resilience regulatory and evidence messages, as well as highlighting the potential role of complexity tools in strategy formulation. Animations are presented showing the dynamics of resilience communication, which is discussed. Reflections on the use of participatory social network analysis are explored, as the method opens new doors to potentially examine how network changes could alter communication. Key insights highlight that perceived responsibilities for resilience in the UK water sector rest with a small core of organisations; water customers play a limited role in the two-way communication of resilience and water sector organisations do not communicate widely on resilience with other sectors (such as energy). Additionally, who an organisations’ neighbours are and what catalyses a message to be passed are important in determining how quickly messages spread. Results lead to a recommendation that high level governmental and policy organisations should engage to a greater extent with new resilience knowledge and consider the use of complexity tools in policy making. Policy in relation to resilience is not keeping pace with such knowledge, limiting the communication and learning of organisations who ardently follow policy and regulation. For inter-organisational cooperation to make a difference to water governance, such organisations need to be encouraged to communicate and embed the latest approaches in relation to resilience and complexity thinking and practice.

## 1 Introduction

In social-ecological systems (SES), resilience is a long-standing, well-defined concept that has been explored and assessed using numerous methods and across a range of disciplinary cases (Carpenter et al., 2001; Folke et al., 2010, 2016). Additionally, Social Network Analysis (SNA) has emerged as a tool to explore the structures and dynamics of networks across a range of SES, as well as their resilience (DeBresson and Amesse, 1991; Janssen et al., 2006; Rathwell and Peterson, 2012; Tobin et al., 2014; Hauck et al., 2016; Rockenbauch and Sakdapolrak, 2017; Salpeteur et al., 2017). Increasingly, SNA has also focused on water governance across a range of contexts, including actors influencing water flows in Tanzania (Stein et al., 2011); access to water-related education in Arizona, USA (Cutts and Munoz-Erickson, 2015); governance transitions in the Klamath river basin, USA (Chaffin et al., 2016); water-sanitation (WatSan) non-governmental organisation exit strategies in Nicaragua (Walters, 2016); stakeholder interactions in Malta (Gatt, 2016); floodplain management in the Dutch Rhine delta (Fliervoet et al., 2016) and water management in a mining company (Kunz et al., 2017). Water governance represents the interface between natural resources such as water and energy and how they are processed, conveyed and managed for use within societal practices such as cooking, showering, recreation and their industrial parallels (Browne, 2015). This is where SES must broaden out to encompass the ‘technical’: where technologies and infrastructures represent a direct connection between social and ecological systems, social-ecological-technical systems (SETS) thus emerge (Ward and Butler, 2016).

The developing field of resilience engineering and assessment of SETS is beginning to follow that of SES, where a range of quantitative resilience measures for water infrastructure systems have been forthcoming from a range of international disciplinary perspectives (for a comprehensive review refer to Shin et al., 2018). Whilst there is a growing literature on resilience and the analysis of physical water infrastructure systems using network analytics (water distribution systems (Diao et al., 2014, 2016; 2017; Candelieri et al., 2015)), sewer and drainage networks (Juan-García et al., 2017; Mugume and Butler, 2017), the application of SNA to water policy, management and governance-focused organisational infrastructures is only beginning to emerge (Ward and Butler, 2016). This paper outlines a resilience-focused water governance SNA at the inter-organisational level to expand this growing area and provide suggestion for future research directions. Focusing on the UK water sector, the motivation for the research was to explore the utility of complexity tools such as SNA using more participatory approaches in both resilience analysis and policy and decision making. The paper proceeds as follows. The next section briefly outlines the case for using more participatory approaches to SNA (‘PSNA’). The following section describes the PSNA undertaken and details the development of a new SNA resilience communication method and visualisation technique. The penultimate section describes the results of the application of the new method, which is followed by a Discussion and Conclusion section.

## 2 Caution: social network ahead - stepping inside the UK water sector organisational network

Scott (2015) asserts the need for ‘stepping inside’ (pg 459) SNA through ethnographic and reflexive research in order to respond to contestations over its use orienting around its positivist roots. Additionally, there is the need to reduce the risk that a network model replaces reality, becoming the focus of management (Rayner, 2012). Past approaches to SNA have quantitatively examined personal relations of technology inventors in Germany (Cantner and Graf, 2006), explored and quantified the role of informal relationships in influencing organisational change (Barchiesi et al., 2008), analysed knowledge flows within a virtual learning environment (Pasqualino et al., 2012) and utilised mixed methods to interrogate knowledge networks and the transfer of advice among corporate inventors (Brennecke and Rank, 2017). However, it is vital to be aware of issues of power, financial biases and rigid institutional associations in the pursuit of understanding social networks and the impact of changes in structure on their functioning (Rayner, 2012). Furthermore, as SNA has led to a focus on resilience in SES, resilience itself has been criticised as functionalist, presenting the assumption that there is already or could be agreement on a desired end state of an unchanging social system (Brown, 2014).

Taking these issues into consideration it is important to recognise that the water sector in the UK represents a policy context where the institutional enmeshing of commodity-like entities such as water are often removed from being entirely managed or governed at the local scale due to marketization, making the operationalisation of resilience thinking a complex one. In the UK a mixture of public-private (Scotland, Northern Ireland), private (England) and employee-owned (Wales) companies provide water, wastewater and stormwater services under regulated and non-regulated regimes. These four models of operation and the rise of resilience in overarching policy and regulation over the last six years (Ofwat, 2017) place the UK in a unique position to act as a case study through which to examine resilience in action. All countries operate within the EU Water Framework Directive (though this is under review as a consequence of Brexit) and as several basins cross borders between countries, there are obligations for organisations to work together in water management. In England and Wales, policy and regulation is usually set by the English government, with the Welsh government adapting it into its own legislation, as in the case of the Environment Agency and Natural Resources Wales, and the Drinking Water Inspectorate for environmental and health regulation, respectively. Ofwat (the Water Services Regulation Authority) provides economic regulation for both England and Wales. Resilience entered water management legislation for England and Wales in 2014 through the Water Act 2014 (‘The Act’), intended to explicitly update the Water Industry Act 1991. Ofwat has the primary duty to secure resilience (‘The Duty’) and to embed resilience in Water Resources Management Plans. However, in parallel with this the English Government's Department for Environment, Food and Rural Affairs developed the ‘Creating a great place for living: enabling resilience in the water sector’ roadmap for securing resilience (Defra, 2016).

In this complex landscape, networks comprise governmental, semi-governmental (‘quango’), non-governmental, regulated, independent and other formal private sector organisations, as well as a layer of civil society, informal and more local interest groups (Ward and Butler, 2016). Consequently, the research presented here proceeds with caution in its contribution to ‘stepping inside’ a network through the use of a more reflexive approach, acknowledging that whilst SNA has its strengths it also has its limitations.

Subsequently, examination of a particular conception of inter-organisational resilience in the water sector in the UK is proposed here. By inter-organisational resilience we mean the ability of the organisations within a network to share knowledge and communicate to ensure the network responds to and recovers from change in a way that minimises the magnitude and duration of any network failure (such as a change in knowledge or an organisation no longer being a member of the network, for whatever reason) (Butler et al., 2016). A pragmatic approach to the method is taken, recognising that a plethora of recent SNA studies have explored the use of quantitative-qualitative methods (Stein et al., 2011; Fliervoet et al., 2016), participatory SNA (Hauck et al., 2016; Ward and Butler, 2016; Kunz et al., 2017) and other combinations such as semantic and SNA using social media platforms such as Twitter (Bunney et al., 2018; Barchiesi and Colladon, 2019). Taking resilience as the starting point, an assumption is made that regulation and evidence dominate inter-organisational social relations at the sectoral level (rather than market-driven prices or commodities as in Scott's (2015) due to the way in which the UK water sector is regulated). Scott's assertion that connectivity does not necessarily induce communication and consensus is also considered through ethnographic reflection on knowledge of modes of communication (represented by edges) between individuals with duties relating to resilience in water sector organisations (represented by nodes). Finally, there is no consensus building aim in the method presented; multiple PSNAs were derived and analysed and therefore use is made of the ‘clumsy’ arrangements that are usually removed as uncomfortable knowledge in SNA studies (Rayner, 2012).

## 3 Conceptualising a resilience-focused water sector organisational network

**Figure 1:**
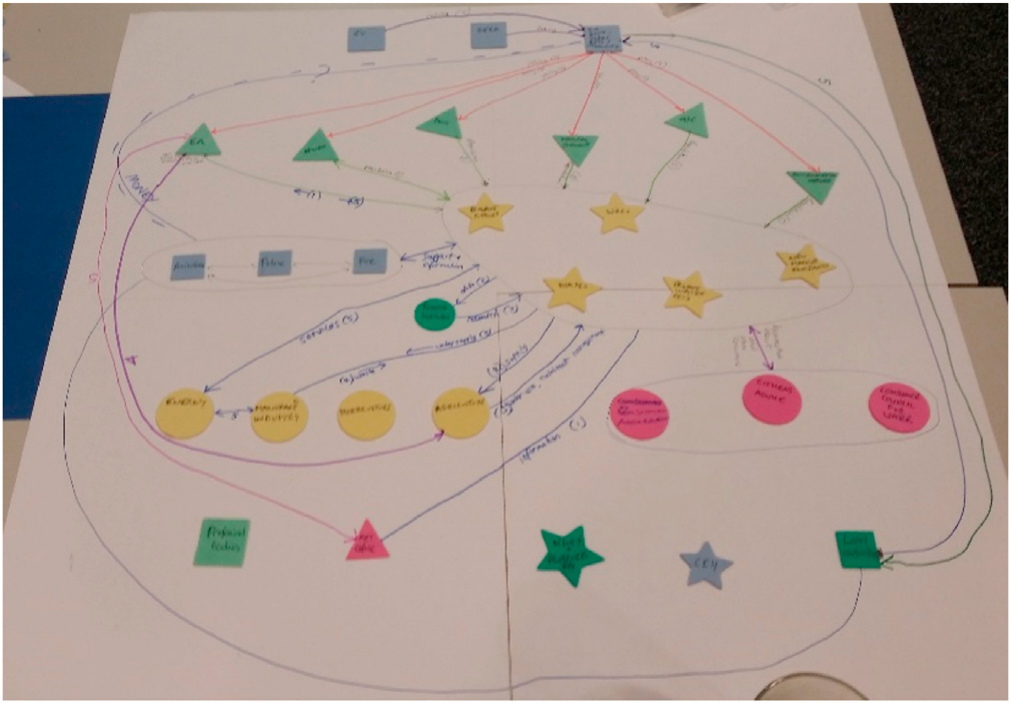
An example of a participatory social network representation of organisations with roles relating to resilience in the UK water sector.

**Figure 2:**
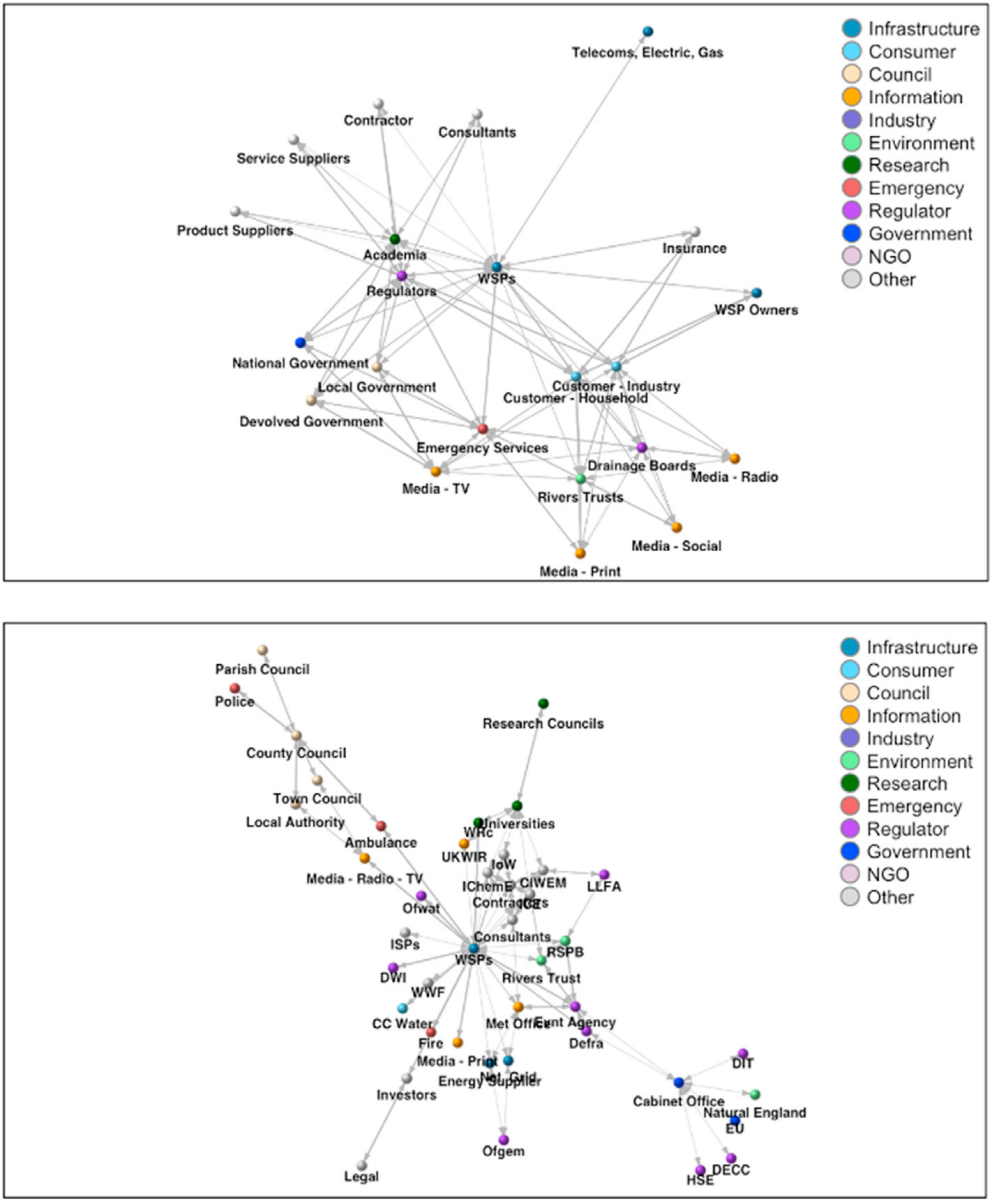
Examples of digitally coded *weighted* participatory social network representations of organisations with roles relating to resilience in the UK water sector.

Unlike in most SNA research, where quantitative data is obtained through databases (Binz and Truffer, 2011) or via a researcher/research team defining actor-network ties and boundaries, with possible reflection (Prell et al., 2009; Stein et al., 2011; Ward and Butler, 2016), participatory SNA (PSNA) begins with workshops, where a range of representatives are gathered together to explore their conceptions of a network and to identify such actors, ties and boundaries. In this research the Steering Group for the Safe and SuRe research project (http://safeandsure.info/) provided an unparalled opportunity for the elucidation of networks representing organisational interactions around resilience in the UK water sector. Comprising high-level representatives of a range of organisations (from water companies to regulators to independent consultants), the Steering Group (‘the Group’) met with the Safe and SuRe project team twice a year to discuss project progress and state of the art research on tools to assess resilience in water systems. Each meeting was regularly attended by between eight and twelve individuals. During one of the meetings the project team presented the Group with the opportunity to formulate networks representing organisations with roles or responsibilities for resilience in the water sector in the UK. Roles and responsibilities could range from regulation to research, evidence synthesis to information sharing or awareness raising – any activity the Group deemed appropriate to contributing to the Resilience Duty of the Water Act 2014 (discussion of the evolution of this legislation is beyond the scope of this paper). The Group were split across four tables to facilitate this, as it was understood that consensus was unlikely to be reached amongst the whole larger group. Each table was provided with large coloured card squares, colouring pens, coloured foam stickers and other materials to creatively construct a representation of the network (e.g. Figure 1). Each member of the Group used their knowledge of the sector and relations and all communications (edges) therein to link water sector organisations (nodes). They were also asked to weight the edges to represent the strength of the links between the nodes (1 = low strength; 5 = high strength). The Group was briefed that the outputs they produced would be coded into digital network maps (e.g. Figure 2; produced using the igraph package in R), which would be shown back to them and then interrogated using SNA tools. Additionally, in order to interrogate the network with regard to organisational interactions in relation to resilience, a new approach was developed, which is described in the following section. Figure 2 network maps illustrate the Groups' representations of the two-way relations amongst the organisations they perceived to have a role in or responsibility relating to resilience in the UK water sector. Some organisations appear in both diagrams as different tables (sub-groups of the Group) created them. This suggests there is consistency across water professional's perceptions of the organisations with a role or responsibility for resilience. However, the differences between the two networks highlight that there are also inconsistencies in the way the relations amongst the organisations are perceived, which could have implications for communication across and hence the inter-organisational resilience of the network.

## 4 Conceptualising resilience communication amongst water sector network organisations

Using the information exchange methodology developed for theoretical networks by Tran et al. (2017), a method was developed to represent communication as message passing throughout the water sector organizational network. This is in contrast to the advice ties method utilized by Brennecke and Rank (2017), as that assumes that a node is actively seeking advice rather than messages circulating more generally within a network and only represents ties in an adjacency matrix rather than stepping further into the network through animating the results. In this research, the passing of two types of resilience message was modelled: (i) regulation and (ii) evidence. It was assumed that the concept of resilience was fully engaged with by an organization and could be spread further only if a node received both the regulatory and evidence messages. Otherwise, a node would pass on the single type of message it received. The visual network graphs provided back to the Steering Group and results from graph theory metrics, such as betweenness and degree centrality, were produced using the igraph package in R, which is an accessible environment for statistical computing and graphics. Code for the simulation of resilience communication was, however, developed in Matlab (though other programming languages such as R can also be employed) according to the description of the two methods for message passing reported in Tran et al. The main reason for the use of the two programming approaches was different researchers worked on these aspects of the research and the choices reflected their expertise and preferences. The reader can contact the authors if they are interested in acquiring a copy of the code for education or research (i.e. non-commercial) purposes. A graph was developed to describe the relationships between different organizations, where a node represents an organization and a link between two nodes shows that there is a connection between the two organizations. An (square) adjacency matrix was created as a mathematical representation of the graph. If an element in the adjacency matrix is one, it means there is a connection between the two organizations represented by the corresponding row and column; if the element value is zero, it means there is no connection between the corresponding two organizations. These metrics are explored further in the interpretation sections. Weights were assigned to nodes to show the nodal status (e.g. active or inactive). Message passing starts from active node(s) and spreads to all of its neighbours. It is assumed that the speed of message passing is the same, i.e. one connection per time step (though this can be weeks to months, which is case specific). Another matrix is developed to record the activation status of all nodes in the network. However, message delivery does not necessarily lead to engaging with the message as explained further below.

In order to create a dynamic representation of how organisations might pass such resilience messages (on regulation and evidence) through the network, animations were created in Matlab based on the following assumptions across two new methods. Two methods were used in order to compare their impact on the dynamics of communication across the networks:

• Certain nodes create and receive messages: regulatory bodies (which refer to Ofwat in this work as it is the financial regulator with major responsibility for furthering the Resilience Duty) create regulatory messages and research organizations create evidence messages. All stakeholders in the water sector network can receive and pass messages as communication between two connected nodes is considered to be bidirectional (as per the directions indicated by the Group).
• If there is an edge between two nodes in the network, messages spread subject to a probability (weight determined by Steering Group × 0.1) to represent the likelihood of communication. By doing so, it is assumed that the progress of message passing is mainly determined by the stochastic nature of communication and social acceptance rather than the actual speed of message delivery between two organizations.
• Node status is checked at each time step to identify whether messages have been received and whether the concept of resilience has been understood and engaged with (indicated by receiving both types of message).
• The evolution of the spread of messages is observed, especially how and when messages are delivered to water consumers and when the concept of resilience is engaged with.

A second method of simulating message passing through the network was developed based on the Diffusion of Innovation/influence linear threshold model (Kempe et al., 2003) and comprises the following steps:

• It is assumed that the resilience message (no differentiation between evidence and regulatory messages) spreads from a node in the network (e.g. university). The value of nodes which have engaged with the concept of resilience is 1; otherwise, the nodal value is 0. As such, the value of the starting node is 1.
• The resilience message is spread in faith by imitations and a neighbouring node engages with the resilience concepts until the influence from its neighbours is strong enough. To simulate this process, the influence of a node spreading the message to its neighbours is assigned a value determined by the relative level of influence (represented by link weight, determined by the Steering Group) of the spreading node against other neighbours of the neighbouring node. For example, the influence to a neighbouring target node is 0.2 if the weights of all links with which the neighbouring node is connected are 1 (connection with the spreading node), 3, and 1.
• The influence of all neighbouring nodes can add up at each time step. If the total influence value reaches beyond a predefined threshold (e.g. 0.3), the node is active (i.e. it engages with the resilience concept) and its value is set as 1 for the next time step (i.e. it can start spreading resilience messages afterwards). Otherwise, the value of the node is reset as 0.
• The evolution of the passage of resilience messages is observed until all nodes are active.

Whilst not assessed in this study, the implications of variable message creation were also considered (for example, varies at a particular rate e.g. 3 vs 7 messages entering the network per time step (low vs high flow)). Animations for each run of the Matlab model are presented in Appendix 1 and discussed in the following section.

## 5 Visualising resilience communication

Bringing together the PSNA and the message passing methods described in the previous sections enabled the water sector organisational network to be interrogated using standard SNA metrics and visually through the creation of graphs and animations (Appendix 1).

### 5.1 Interrogating water sector organisational PSNA metrics

To examine the consistency and inconsistency of the sub-group networks in comparison with each other, quantification of the number of nodes, number of node groups (self-determined by the sub-groups) and number of edges for each network were calculated. These are presented in Table 1, which highlights that whilst the number of node groups is consistent, there is some inconsistency in the number of nodes and edges. Two of the sub-groups broke down some node groups into multiple nodes (e.g. ‘emergency services’ in one network may have been represented as ‘fire’, ‘ambulance’ and ‘police’ in another network), which explains some of the inconsistency in node and edge numbers. In addition to this, one sub-group's network had isolated nodes with no edges (T2) and another sub-group's network included multiple edges where they perceived there to be more than one method of communication between the organisations represented by the nodes (T1), which led to lower and higher edge counts, respectively. This has perhaps the most potential to influence the communication of resilience messages within the network, which will be discussed further in following sections.

**Table 1:** Consistency/inconsistency across co-produced networks quantified through node, node group and edge numbers.

Node Group	Network Reference			
	T1	T2	T3	T4
WSPs	1	1	1	1
Regulators	1	1	1	1
Local Govt/LA	1	1	1	1
Devolved Govt	1	0	0	0
National Govt	1	1	1	1
Quangos	0	1	1	0
EU	0	1	1	1
OCED	0	0	1	0
Media (all)	1	1	0	0
NGOs/Charities	1	1	1	0
LLFAs/Drainage boards	1	1	0	1
Emergency services	1	1	1	0
Product/service suppliers	1	1	1	1
Contractors/Consultants	1	1	1	1
Customers	1	0	0	1
Customer Challenge Groups	0	0	0	1
Academia/Research Institutes	1	1	1	1
WSP Owners/Investors	1	1	0	1
Insurance/Legal	1	1	0	0
Professional bodies	0	1	1	0
Met Office	0	1	1	0
Horticulture/Agriculture	0	0	1	0

**Total no. node groups in each network**	**15**	**17**	**15**	**12**
**Total no. of nodes in each network**	**22**	**42**	**26**	**20**
**Total no. of edges in each network**	**140**	**110**	**76**	**57**

Centrality measures of degree and betweenness were calculated for each network developed by the Group and are summarised in Table 2. Degree centrality was calculated to identify organisations with the greatest number of perceived direct connections to other organisations in the network (Meng et al., 2018). These ‘highly connected’ organisations occupy a more central position within the network and may influence communication and the passing of messages because they possess a greater number of direct connections with other organisations.

**Table 2:** Resilience-focused water sector organisations with the greatest degree and betweenness centrality for each network co-produced with the Steering Group.

Network T1	Degree		Betweenness
Water Service Providers	36	Water Service Providers	146.20
Regulators	22	Customer - Household	36.65
Customer - Household	22	Customer - Industry	36.65
Customer - Industry	22	Regulators	36.11
Academia	20	Emergency Services	32.48
Network T2	Degree		Betweenness
Water Service Providers	50	Water Service Providers	1326.92
Cabinet Office	14	Cabinet Office	380.00
Universities	14	Environment Agency	245.09
Consultants	14	Ambulance	210.00
Environment Agency	12	Defra	169.08
Network T3	Degree		Betweenness
Water Service Providers	34	Water Service Providers	270.83
UK Government	24	UK Government	124.5
Ambulance	10	Environment Agency	18.5
Police	10	Ambulance	11.83
Fire	10	Police	11.33
Network T4	Degree		Betweenness
Water Service Providers	27	Water Service Providers	259.16
Defra/National Government	13	Defra/National Government	106.17
UKWIR	6	Environment Agency	47.95
Environment Agency	6	DWI	25.08
Local Authority	6	Water UK	25.08

For each table's network, Water Service Providers (WSP's) were perceived to have the greatest number of direct connections with other resilience-focused organisations and occupied a more central position within each network. However, there was a great deal of variation between each network regarding the perception of other ‘highly connected’ organisations. For instance, in Network T1 (Table 2), the WSP's, Regulators, Customers and Academics were perceived to be ‘highly connected’. Whereas in Network 3 (Table 2), WSP's, UK Government, Ambulance, Police and the Fire Brigade were perceived to be ‘highly connected’. Each set of data forms a long-tail distribution pattern that is similar to the others. This pattern, observed in many networks, on the one hand reveals that only a few organisations dominate the network, but on the other hand reveals self-similarities of different properties, e.g. degree and betweenness centrality in this case (Diao et al., 2014, 2016; 2017; Meng et al., 2018).

Betweenness centrality measures the extent an organisation (node) lies on the shortest path between other organisations (nodes) within the network and, as with degree centrality, can be used to assess resilience based on topological characteristics of a network (Meng et al., 2018). Organisations with a high betweenness may influence the passing of messages between other organisations. The removal of an organisation considered ‘influential’ may therefore prevent message passing by lying on the greatest number of ‘shortest paths’ through the network. For each network, WSP's had the greatest betweenness centrality and by lying on the greatest number of shortest paths through the network have the potential to influence message passing throughout the network. There was less variation between each network regarding the other organisations with a high betweenness. For example, the UK Government; Regulators such as Defra and the Environment Agency; and the Emergency Services were included within each network.

By identifying the nodes with a high degree and betweenness centrality, it would be possible to assess the how the purposive removal or reconnection of an organisation might result in larger impacts on message passing than just random node removals. However, Tran et al. (2017) highlight that there is minimal difference in the recovery of a network between random reconnections and preferential ones i.e. whether reconnections occur as the result of purposive change or an interruption from an unknown source, the difference in the effect on the recovery of the network is minimal. Either way, such further analysis is beyond the scope of this paper.

### 5.2 Examining and animating resilience communication amongst water sector network organisations

**Figure 3:**
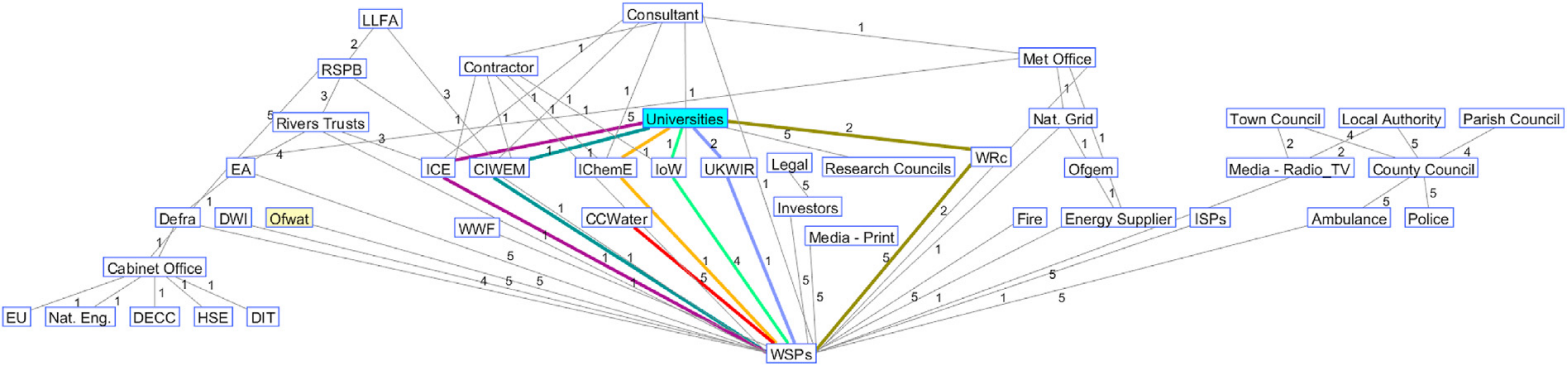
Representation of how resilience messages pass through the UK water sector organisational network (Network T2) (link weight marked numerically on lines representing the links; red line represents the link between CCWater and WSPs; lines presented in colours other than black and red show links between universities and WSPs).

**Figure 4:**
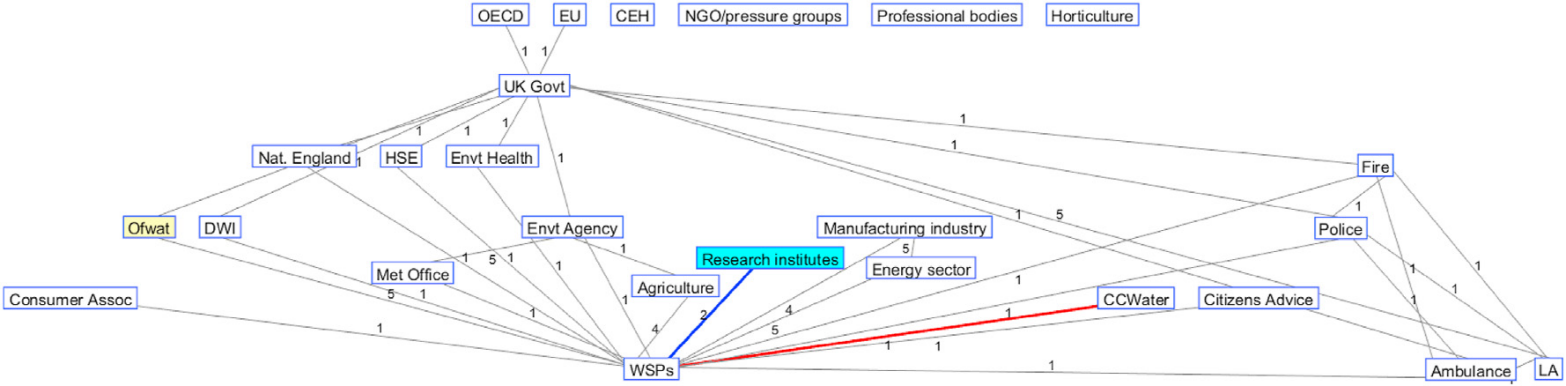
Representation of how resilience messages pass through the UK water sector organisational network (Network T3) (link weight marked numerically on lines representing the links; red line represents the link between CCWater and WSPs; blue line shows the link between research institutes and WSPs).

Taking the interrogation outlined in the previous section further, visualisation of message passing was explored through the creation of graphs and animations (Appendix 1) showing stages of message passing from source nodes (financial regulator and research organisations) to target nodes (water customers). For example, Figures 3 and 4 show the shortest paths between source organisations and target organisations using the network of water sector organisations developed by the second and third tables of the Group (Networks T2 and T3, respectively). For Network T2, the evidence messages generated by Universities can only reach the Consumer Council for Water (CCWater, a statutory consumer body for the water industry in England and Wales, representing the water customer in this network) via Water Service Providers (WSPs) (red line). There are six shortest paths from Universities to WSPs as highlighted in purple, mint, orange, green, blue and moss green lines. Regulatory resilience messages are passed to CCWater also via WSPs. Due to the stochastic nature of the message passing and engagement, only the Institute of Water (IoW) (the assigned weight value of the link with Universities is 1), the Institution of Civil Engineers (ICE) (weight: 1) and the UK Water Industry Research (UKWIR) (weight: 2) receive the evidence message from Universities at the end of the first time step. The Chartered Institution of Water and Environmental Management (CIWEM) (weight: 1), the Institution of Chemical Engineers (IChemE) (weight: 1) and the Water Research Centre (WRc) (weight: 2) do not receive the message until later.

**Figure 5:**
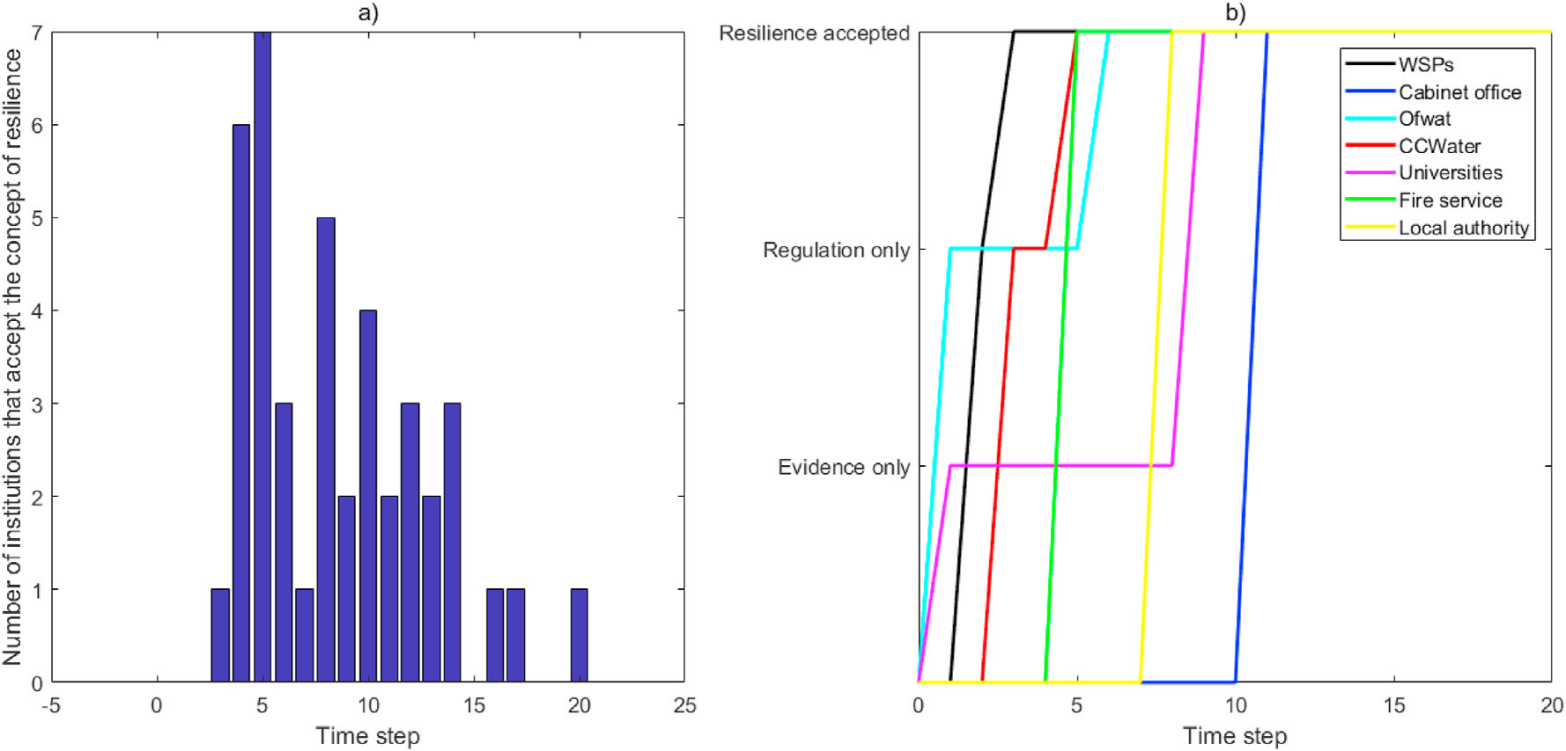
a) Number of institutions that engage with the concept of resilience at different time steps; b) Message pathways for selected institutions in Network T2.

Another way of viewing the progress of message passing and engagement with the resilience concept is presented in Figures 5a and 5b. Engagement with the resilience concept varies among the organizations in the network as illustrated in Figure 5a, where communication pathways across time steps differ as the two message types are passed and received at different rates by different nodes. This is further illustrated by selected organisations in Figure 5b. For example, WSPs and CCWater receive regulation-related resilience messages first and fully engage with the resilience concept one or two time steps later after they receive the evidence messages; fire service, local authorities and the Cabinet Office engage with the resilience concept influenced by neighbours that have fully engaged with the resilience concept. Figure 5b shows that after Universities produce evidence messages and Ofwat produces regulatory messages, they reach WSPs and CCWater fastest, but Local Authorities and the Cabinet Office slowest. This is surprising as the Cabinet Office (2011) provided some of the earliest guidance on which the majority of the water sector currently bases its approach to resilience, but is unsurprising in that it has not updated that guidance since that time. The delay in Local Authorities receiving both types of message could explain their uncertain role in multi-agency emergency planning and connectedness through social media platforms (e.g. Twitter) and representation on Local Resilience Forums where they are expected to liaise with WSPs (Bunney et al., 2018). In both these cases there are potential interventions that could be recommended, such as the Cabinet Office strengthening links with Universities or intermediary organisations to ensure that evidence messages are passed back to enable refreshing of policy resilience with new knowledge. Barchiesi and Colladon (2019) investigated Italian firms' discussion of core values through semantic and SNA of Twitter posts, highlighting that firms’ core value orientation could be elucidated through SNA. They further assert that big data and complexity analysis tools should be integrated into strategy formulation and implementation processes in complex business environments – the same could be asserted for the policy and regulatory environments, such as the UK water sector, based on the results of the SNA in the present paper.

The dynamics of message passing were explored further through the creation of animations (Appendix 1) showing the movement of messages from source organisations to target organisations. This enabled differences in the spread of message passing to be directly comprehended. File T2 shows the animation for the table two network without the influence of the diffusion of innovation (DoI) weighting, whilst file T2-innovation shows the animation with the influence of the DoI weighting. The same scenarios are represented in files T3 and T3-innovation for table three. When message passing begins, inactivated organisations are represented in white, organisations receiving regulatory messages become beige, organisations receiving evidence messages become blue and when organisations receive both message types and engage with the resilience concept they become green.

For Network T2 at time step (Ts) 1, Universities are blue (passing evidence messages), Ofwat is beige (passing regulatory messages), CCWater and Environment Agency (EA) are beige (receiving regulatory messages), IoW (Institute of Water), ICE, Research Councils and Rivers Trusts are blue (receiving evidence messages) and WSPs are green (received both message types and engage with the resilience concept). At Ts5, as well as WSPs the following organisations have become green: IoW, WRc, the Met Office, Defra, EA, Rivers Trusts, the Royal Society for the Protection of Birds (RSPB), Drinking Water Inspectorate (DWI), World Wildlife Fund (WWF), CCWater, Investors, Media, Emergency Services (Fire, Ambulance) and County Councils. Consultants and the National Grid are beige and ICE, Research Councils and UKWIR are blue, with all remaining organisations white. It is not until Ts19 that all organisations become green, with Ofgem and the Dept. for Trade and Industry (DTI) being the last to receive both types of message and engage with the resilience concept. This indicates that the dominant organisations are connected through resilience, but that water sector organisations do not communicate widely on resilience with other sectors (such as energy), as perceived by the Steering Group.

Comparing these observations with T2-Innovation, the differences are that at Ts5 only the Cabinet Office (and associated depts.), the EU, Natural England, Legal, Police, all types of local Council and Ofgem have not become green. However, all organisations are green by Ts7. This indicates that the DoI method increases the rate at which messages are passed through the network, suggesting that who an organisations’ neighbours are and what catalyses a message to be passed are important in determining how quickly messages spread through the network. Steering Group members and other water professionals were shown the animations and asked for their input on how they could be useful to their organisations and the sector as a whole in resilience decision or policymaking. They were interested to know, for example:

• What differences are there when message source organisation and target organisation change?
• How would results change if links perceived by all tables remained the same, but links perceived differently changed (i.e. reconnected with a different organisation)?
• What would change if certain links were weaker or stronger? (representing an increased number of methods of communicating perhaps).

Whilst these are all valuable questions and worthy of investigation as part of further research, their value in the real world is questionable. Whilst it is possible to alter how links are connected, the strength of links and message passing rates in the network representations, to do so with actual water sector organisations is unrealistic due to layers of legislation, regulation, policy and the logistics of practice. However, the main implication of this analysis for water policy is that the use of complexity tools could provide the ability to show decision and policy-makers where they could perhaps make greater efforts to communicate with particular organisations in order to more efficiently increase the interchange of knowledge on resilience within and between sectors.

## 6 Reflections and conclusions for water sector inter-organisational network resilience

The PSNA and novel method presented in this paper highlight that perceived responsibilities for resilience in the UK water sector are situated firmly with a small core of organisations, representing polycentric governance with water customers playing a limited role in the two-way communication of resilience. If we consider extreme events requiring enhanced resilience, there exists a tension between governance where responses are currently more decentralized across a number of organisations and encourage local action in more group-orientated ways (e.g. for flood events) and where responses are currently more centralized to WSPs and encourage individual rather than collective action (e.g. for drought events). Potentially in either case, if the customer is expected to participate in capacity building for resilience, their position in the network may need to be more central. Therefore it is difficult to identify whether a more neoliberal participatory or macro-regulatory overhaul (or somewhere in between) may be warranted to enhance inter-organisational resilience in the UK water sector.

Responding to the call for more self-reflective studies, the research aims to not reinforce anything other than a considered stance on the results and representations and the insights they provide, hence demonstrating caution in making suggestions for network governance optimization. However, in relation to water policy, one recommendation made based on the strength of the results is that high level governmental and policy-influencing organisations should engage to a greater extent with new knowledge in order to update policy to ensure those organisations following policy and regulation are in possession of the latest approaches in relation to resilience thinking and practice. This has implications for UK water policy as currently the use of PSNA and other complexity tools in strategy formulation and policy change implementation processes is not on the agendas of or within the organizational cultures of most organisations within the network or water sector as a whole. The current focus on big data and Internet of Things approaches is for technical/operational projects rather than business-side change management, so a shift to include application for resilience in strategy and policy is needed.

Consequently, whilst the application of PSNA to a water governance issue has revealed useful insights for the inter-organisational resilience of the UK water sector, the research presented illustrates, in conclusion, that:

• Betweenness and degree centrality are useful metrics, but do not enable full observation of the dynamics of communication between organisations;
• There is value in visualizing message passing and communication across an organizational network to observe how organisations receive and pass on messages;
• Whilst these observations are insightful and artificially manipulating reconnections in the network is possible, there may be limited value in doing so as replication of the ‘most efficient’ organizational network in the real world is likely to be challenging;
• Further analysis of such networks could enable decision and policy-makers to identify where greater communication is required to maintain or enhance inter-organisational resilience;
• A shift is required to better embed the application of complexity tools in examining organizational and policy challenges for resilience as well as operational challenges.

Other work in the area of understanding network communication is encouraged, for example through the use of PSNA applied in other resource sectors, to enable the debate to continue on the use of SNA as a whole in the area of policy, technology and innovation, particularly with a focus on resilience.

## Declarations

### Author contribution statement

S. Ward, F. Meng and S. Bunney: Conceived and designed the experiments; Performed the experiments; Analyzed and interpreted the data; Contributed reagents, materials, analysis tools or data; Wrote the paper.

K. Diao and D. Butler: Conceived and designed the experiments; Wrote the paper.

### Funding statement

This work was supported by the UK Engineering & Physical Sciences Research Council (EP/K006924/1 (Safe & SuRe Water Management)).

### Competing interest statement

The authors declare no conflict of interest.

### Additional information

No additional information is available for this paper.

## Appendix A Supplementary data

T1_V2 T1_V2

T1-innovation_V2 2 T1-innovation_V2

T2_V2 3 T2_V2

T2-innovation_V2 4 T2-innovation_V2

T3_V2 5 T3_V2

T3-innovation_V2 6 T3-innovation_V2
